# Polypharmacy and adverse outcomes after hip fracture surgery

**DOI:** 10.1186/s13018-016-0486-7

**Published:** 2016-11-24

**Authors:** Maria Härstedt, Cecilia Rogmark, Richard Sutton, Olle Melander, Artur Fedorowski

**Affiliations:** 1 Department of Clinical Sciences, Malmö, Faculty of Medicine, Lund University, SE 205-02 Malmö, Sweden; 2 Department of Orthopaedics, Skåne University Hospital, SE 205-02 Malmö, Sweden; 3 National Heart and Lung Institute, Imperial College London, St Mary’s Hospital Campus, 59-61 North Wharf Road, London, W2 1LA UK; 4 Department of Internal Medicine, Skåne University Hospital, SE 205-02 Malmö, Sweden; 5 Department of Cardiology, Skåne University Hospital, Inga Marie Nilssons gata 46, SE 205-02 Malmö, Sweden

**Keywords:** Hip fracture, Patient readmission, Mortality, Polypharmacy

## Abstract

**Background:**

We aimed to explore the effects of polypharmacy and specific drug classes on readmissions and mortality after hip surgery.

**Methods:**

We analyzed data on 272 consecutive hip fracture patients (72.1% females; age 82 ± 9 years) who underwent acute hip replacement. We collected detailed data on the pharmacological treatment upon admission and discharge. Patients were followed up over a period of 6 months after discharge using the Swedish National Hospital Discharge Register and the Swedish National Cause of Death Register.

**Results:**

After 6 months, 86 patients (31.6%) were readmitted, while 36 patients (13.2%) died. The total number of medications upon discharge was predictive of rehospitalization (odds ratio (OR) 1.08, 95%CI 1.01–1.17, *p* = 0.030) but not predictive of mortality. The use of antiosteoporotic agents (OR 1.86, 95%CI 1.06–3.26, *p* = 0.03), SSRIs (OR 1.90, 95%CI 1.06–3.42, *p* = 0.03), and eye drops (OR 4.12, 95%CI 1.89–8.97, *p* = 0.0004) were predictive of rehospitalization. Treatment with vitamin K antagonists (OR 4.29, 95%CI 1.19–15.39, *p* = 0.026), thiazides (OR 4.10, 95%CI 1.30–12.91, *p* = 0.016), and tramadol (OR 2.84, 95%CI 1.17–6.90, *p* = 0.021) predicted readmissions due to a new fall/trauma.

**Conclusions:**

The total number of medications, use of antiosteoporotic agents, SSRIs, and eye drops predicted rehospitalization after hip fracture surgery, while use of vitamin K antagonists, thiazides, and tramadol was associated with readmissions due to a traumatic fall.

**Trial registration:**

Hip fractures and polypharmacy in the elderly. Stimulus Project for the Elderly 2009-2011 (Reg no 2009-11-26). Swedish National Board of Health and Welfare.

## Background

Hip fracture in the elderly, following the trend in population aging in developed countries, is a common cause of hospital admission [1–4]. The mean age of typical patients with acute hip fracture is high, over 80 years [5], which implies multiple comorbidities combined with polypharmacy [5–7]. It is estimated that patients who suffer hip fracture take, on average, six different medications, which have been identified as an independent risk factor for falls in the elderly [8, 9]. Moreover, two thirds of these patients take drugs that raise the fall risk [9]. In recent years, there has been a growing interest in optimizing pharmacological treatment in the elderly by introduction of systematic medication surveys [10, 11], but their effects on post-discharge complications are not universally agreed [12, 13]. In parallel, relatively little is known how polypharmacy and treatment with specific drug classes impact readmission rate and short-term mortality after hip fracture surgery. Accordingly, the aim of this study was to explore the potential association of discharge medications with the postoperative readmission and death rate.

## Methods

### Patients

Between November 2009 and June 2011, we enrolled 304 consecutive patients who were admitted to the Department of Orthopaedics at Skåne University Hospital in Malmö, Sweden, with a preliminary diagnosis of hip fracture. Of these, we excluded 32 patients: 23 who did not meet criteria for hip fracture (International Classification of Diseases, 10th Revision (ICD-10) code S72.0-2) i.e., without confirmed fracture during secondary assessment or with a fracture present in another location; 4 who did not undergo surgery; 3 who underwent surgery at another hospital; and 2 for whom the complete dataset was not available. The remaining 272 patients accepted participation in the study.

### Methods

Patient characteristics were recorded after admission including biometric and routine basic clinical data (blood pressure, plasma concentrations of sodium, potassium, creatinine, and hemoglobin), information about current medications, and their indications plus American Society of Anesthesiologists (ASA) Classification of Physical Health grade [14] assessed before hip surgery. In the next stage, two clinical pharmacists contracted for the project and retrieved the actual medication list from the Swedish National Pharmaceutical Register, which holds records of all prescribed drugs with their dispensing dates for all Swedish citizens. The patient’s family, other caregivers, or general practitioner verified the medication list, if necessary. Drugs were classified according to the Anatomical Therapeutic Chemical (ATC) classification system [15], in which the active substances are divided into 14 different groups according to the organ or system on which they act (1st level), and their therapeutic, pharmacological, and chemical properties (2nd–5th level).

The routine reassessment of the basic clinical parameters (blood pressure, electrolytes, creatinine, and hemoglobin) on the 3rd day after hip fracture surgery was performed, and the corresponding data were recorded in the database. Within the first postoperative week, a clinical medication review based on a structured approach previously proposed [16] was performed by a pharmacist and consultant physician in internal medicine (AF). Dosages and indications were checked, unsuitable drugs were withdrawn, and dosages were adjusted, if appropriate. Local (valid for Skåne region, southern Sweden), national, and international guidelines constituted grounds for decision-making, which was reached by consensus between the pharmacist and physician. In selected cases, the pharmacist and physician discussed the relevance of indications with the orthopedic surgeon or the appropriate specialist. The patient’s general practitioner received written information about alterations, as did the patient or family. Patients were followed up over a period of 6 months after discharge from the hospital using a unique personal 10-digit identity number that can be linked to the Swedish National Hospital Discharge Register and the Swedish National Cause of Death Register.

The number and causes of readmissions and death statistics were retrieved from the available hospital records in the study catchment area and appropriate national registers. The emergency department visits that did not result in hospital readmission were not included. The reasons for the readmission were classified according to the main diagnosis in the ICD-10 coding system. Specific categories for readmission were defined as fall injury, cardiac, neurological, psychiatric, surgical, and infection.

### Statistical analyses

Patient characteristics were reported as mean and standard deviation or proportions as appropriate. Proportions of specific drug use upon admission and their changes after medication review were calculated. The total number and reasons for readmissions as well as mortality within the first six months after discharge from hospital were determined. The relations of total number and type of discharge medications with readmission risk or death, as the categorical dependent variables, respectively, were assessed using a multivariable-adjusted (for age and gender) logistic regression model. In a subsidiary analysis, only readmissions due to fall injury, infection, or cardiovascular disease were assessed as a categorical dependent variable. All analyses were performed using IBM SPSS Statistics version 22 (SPSS Inc., Chicago, IL, USA). All tests were two-sided whereby *p* < 0.05 was considered statistically significant.

## Results

Table 1 shows intervention group characteristics including supine blood pressure, plasma concentrations of sodium, potassium, creatinine, and hemoglobin recorded upon admission, and the routine reassessment of the same clinical parameters on the 3rd day after hip fracture surgery. Women were overrepresented (*n* = 196, 72.1%), and the mean age was 82.0 ± 9.0 years, range from 53 to 100 years.

**Table 1:** Clinical characteristics of study population (*n* = 272)

Parameter	Mean/proportion	Std. deviation	Minimum	Maximum
Age, years	82.0	9.0	53	100
Gender, *n*, % females	196 72.1			
*P*-Glucose admission, mmol/L	7.5	2.1	3.4	19.8
*P*-Glucose, 3rd day, mmol/L	9.0	3.2	5.7	16.9
*P*-Sodium admission, mmol/L	138.7	3.4	125.0	147.0
*P*-Sodium 3rd day, mmol/L	137.0	3.5	120.0	146.0
*P*-Potassium admission, mmol/L	3.9	0.5	2.6	5.9
*P*-Potassium 3rd day, mmol/L	3.7	0.5	2.4	5.5
*P*-Creatinine admission, μmol/L	88.5	48.6	26.0	563.0
*P*-Creatinine 3rd day, μmol/L	91.2	67.4	34.0	614.0
*P*-Hemoglobin admission, g/L	122.7	15.7	74.0	192.0
*P*-Hemoglobin 3rd day, g/L	106.2	13.1	72.0	143.0
Blood pressure-systolic admission, mmHg	144	22	90	220
Blood pressure-systolic 3rd day, mmHg	121	18	70	180
Blood pressure-diastolic admission, mmHg	75	12	50	120
Blood pressure-diastolic 3rd day, mmHg	63	10	40	100
No. of medications, admission	6.2	3.9	0.0	20.0
No. of medications, discharge	7.8	3.6	0.0	20.0

Glucose, sodium, potassium, and creatinine were plasma levels

### Medications upon admission and discharge

As can be seen in Table 1, upon admission to hospital patients in the intervention group had on average 6.2 +/− 3.9 prescribed drugs (range from 0 to 20), and the corresponding number was 7.8 +/− 3.6 (range from 0 to 20) upon discharge. Table 2 shows current medications, according to ATC classification, upon arrival at hospital and on discharge in the intervention group. The most common medications upon admission were platelet aggregation inhibitors, antihypertensive agents, and among these, diuretics, beta-blocking agents, and agents acting on the renin-angiotensin system, then, anxiolytics, and antidepressants. The most common medication to be removed or modified during reviews was antihypertensive treatment, discontinued totally in 31 patients. Among the antihypertensive agents, diuretics were discontinued in 39 patients, whereas calcium channel blockers were ceased in 24 patients. Further, there were only slight reductions in the use of beta-blocking agents, hypnotics, sedatives, and antidepressants. There were no changes made in treatment with dopaminergic agents, drugs for obstructive airway diseases, and eye drops. In contrast, low-molecular weight heparin (LMWH, *n* = 89), tramadol (*n* = 147), and paracetamol (*n* = 157) were frequently initiated during hospital stay and prescribed at discharge, whereas antiosteoporotic drugs were initiated in 15 patients during hospital stay.

**Table 2:** Prevalent medications (ATC) upon admission and discharge after hip fracture surgery (*n* = 272)

ATC-code medication	Admission (*n*)	Discharge (*n*)	Difference (*n*)
A02B drugs for peptic ulcer and gastro-oesophageal reflux disease (GORD)	79	82	−3
B01AA vitamin K antagonists	13	12	−1
B01AB heparin group	1	90	+89
B01AC platelet aggregation inhibitors	108	89	−19
C01D vasodilators, (nitrates)	34	40	+6
C03 diuretics, all	113	66	−47
C03A low-ceiling diuretics (thiazides)	35	17	−18
C03C high ceiling diuretics (loop)	72	37	−35
C03D potassium-sparing agents (C03DA, aldosterone antagonists)	31	11	−20
C07A beta-blocking agents	85	84	−1
C08C-D calcium channel blockers	55	31	−24
C09A-C agents acting on the renin-angiotensin system	72	62	−10
C01-09 antihypertensives	179	148	−31
M05B drugs affecting bone structure and mineralization	68	83	+15
N02A opioids strong (excl. tramadol)	14	45	+31
N02AX opioids weak (tramadol)	13	160	+147
N02B other analgesics (incl. paracetamol)	63	220	+157
N04B dopaminergic agents (antiparkinson)	15	15	+/−0
N05B anxiolytics (incl. benzodiazepines)	56	48	−8
N05C hypnotics and sedatives (incl. zolpidem and heminevrin)	80	78	−2
N06A antidepressants, totally	80	79	−1
N06AB SSRI only	66	63	−3
N06D psychoanaleptics (antidementia)	15	14	−1
R03A-B drugs for obstructive airway diseases	28	28	+/−0
S01E-X ophtalmologicals (eye drops)	31	31	+/−0

In summary, upon discharge, the most common medication groups were analgesics, antihypertensive agents, LMWH, antiosteoporotic agents, platelet aggregation inhibitors, and antidepressants.

### Readmissions

During the 6-month follow-up period, 86 patients (31.6%) were readmitted. In total, there were 120 readmissions, and the most common cause of readmission was a new fall/trauma (*n* = 31), followed by infection (*n* = 27). The total number of medications upon discharge was predictive of rehospitalization (OR 1.08, 95%CI 1.01–1.17, *p* = 0.030) as also was plasma potassium on 3rd day post-surgery (OR per one mmol/L 2.74, 95%CI 1.50–5.00, *p* = 0.001), especially in regard to readmission due to a new cardiovascular event (OR 3.36, 95%CI 1.18–9.57, *p* = 0.023), or infection (OR 3.04, 95%CI 1.06–8.71, *p* = 0.039).

**Fig. 1:**
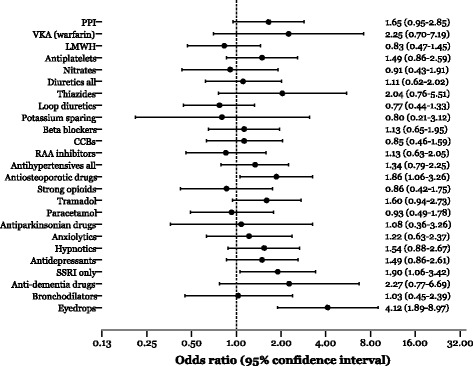
The relative risk of readmission (86/272) within 6 month after acute hip fracture surgery as indicated by specific medication classes upon discharge from hospital in a multivariable-adjusted (for age and gender) logistic regression model

In specific therapeutic groups (see Fig. 1), drugs affecting bone structure and mineralization (=antiosteoporotic agents; OR 1.86, 95%CI 1.06–3.26, *p* = 0.03), SSRIs (OR 1.90, 95%CI 1.06–3.42, *p* = 0.03), and eye drops (OR 4.12, 95%CI 1.89–8.97, *p* = 0.0004) were all predictive of rehospitalization. Further, treatment with eye drops was predictive of fall injury (OR 5.97, 95%CI 2.48–14.33, *p* = 0.00007), as also was treatment with antiosteoporotic agents (OR 2.93, 95%CI 1.34–6.41, *p* = 0.007). In contrast, increased risk of readmission due to infection was associated with SSRI (OR 3.40, 95%CI 1.30–8.88, *p* = 0.013). Other medications associated with increased readmission risk due to a new fall/trauma were vitamin K antagonists (VKA, OR 4.29, 95%CI 1.19–15.39, *p* = 0.026), thiazides (OR 4.10, 95%CI 1.30–12.91, *p* = 0.016), and tramadol (OR 2.84, 95%CI 1.17–6.90, *p* = 0.021). Association between eye drops and fall injury was independent of beta-blocker use as also was association between thiazides and fall injury in regard to plasma electrolytes including potassium.

### Mortality

**Fig. 2:**
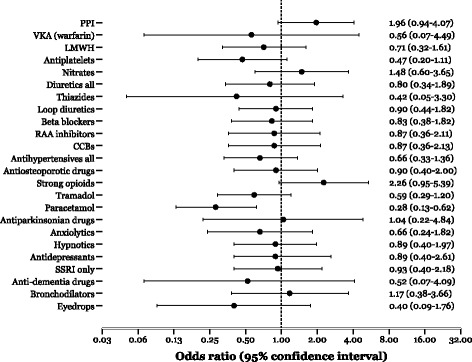
The relative mortality (36/272) within 6 month after acute hip fracture surgery as indicated by specific medication classes upon discharge from hospital in a multivariable-adjusted (for age and gender) logistic regression model. *PPI* proton pump inhibitor, *VKA* vitamin K antagonist, *LMWH* low-molecular weight heparin, *CCB* calcium channel blocker, *RAA* renin-angiotensin aldosterone, *SSRI* selective serotonin reuptake inhibitors

Thirty-six of the 272 patients (13.2%) died within 6 months after hip surgery. The number of medications did not predict mortality (OR 0.97, 95%CI 0.88–1.07, *p* = 0.55). As shown in Fig. 2, the 6-month mortality was inversely associated with paracetamol treatment upon discharge (OR 0.28, 95%CI 0.13–0.62, *p* = 0.001), while there was a tendency for higher mortality among patients treated with strong opioids such as morphine (OR 2.26, 95%CI 0.95–5.39, *p* = 0.065). However, when entered in the same model, the presence of strong opioids in the absence of paracetamol was a significant predictor of mortality (OR 2.95, 95%CI 1.19–7.34, *p* = 0.020), independently of prevalent malignant disease (data not shown).

## Discussion

In this study, we have shown that the number of medications upon discharge is predictive of readmission but not death within six months after acute hip fracture surgery. Further, patients treated with antiosteoporotic agents, SSRI, and eye drops have increased overall risk of readmission, whereas use of vitamin K antagonists, thiazides, and tramadol is associated with increased risk of readmission due to fall injury. Finally, increased mortality may be indicated by a sole opioid use at discharge.

The medication reviews performed in our cohort revealed a considerable number of pharmacological issues that received attention and were resolved, if found appropriate.

Zermansky et al. [17] found consultations with a clinical pharmacist to be effective in care home residents, a study population with mainly the same characteristics as a group with hip fractures. From a practical point of view, this concept worked well in our study; the pharmacist started by interviewing the patient and scrutinized different data sources to complete the medication list. A form was filled in, structuring the review, and the result was presented to the internal medicine consultant who decided by consensus, which changes would be suitable and safe.

Notable was that a new trauma or fall injury were the most common reasons for new contact with the hospital. Prevention of falls, better information regarding the hip fracture, and improvement of rehabilitation seem wise, together with improvement in primary care.

Relationships between discharge medications and post-discharge complications deserve specific comments. The use of eye drops was the strongest single predictor of readmission including new trauma. We suggest that the visual impairment is the decisive factor underlying fall injury, and that this group, although not large in number, should receive more specific care, for instance, through adjustments made in their home environment. Another possible explanation of this observation is induction of bradyarrhythmia by beta-blocking component of eye drops; however, there was no significant interaction between eye drops and beta-blockers.

The association between antiosteoporotic drugs and readmissions due to a new fall injury is more obvious as the elderly with osteoporosis have double the risk of falling compared with those without osteoporosis, not only due to osteoporosis but also due to other shared risk factors such as weight loss, small muscle mass, low muscle strength, low physical exercise levels, and limited mobility [18]. Moreover, low BMI is a factor associated with higher prevalence of orthostatic hypotension [19]. The use of SSRI was particularly predictive of readmissions due to infectious disease. Of these, the two most frequent causes are urinary infection and pneumonia. SSRIs have been reported to cause urinary retention [20] and negatively influence renal and respiratory function in the post-operative period [21]. Moreover, SSRIs are associated with increased risk of falls and confusion as possible contributory factors [22]. The role of VKA in the elevated risk of readmission after fall injury can be explained by medical vigilance when dealing with patients who demonstrate higher probability of occult bleeding, and thus require prompt diagnosis including radiology, laboratory assessment, and extended observation. A fourfold increased risk of readmission due to a new fall injury was associated with thiazides, and not other types of antihypertensive drugs, is remarkable. However, regardless of antihypertensive effect, thiazide diuretics may also be involved in intravascular volume reduction and electrolytic disorders, all of which contribute to the increased risk of falls [23]. These results question the use of thiazide diuretics in elderly and frail patients and suggest the use of other types of antihypertensive drugs, or simply discontinuation of treatment. In parallel, our results question the prolonged use of tramadol in the post-discharge period, as this pharmacological agent, routinely used for the post-operative pain alleviation, was associated with almost threefold increased risk of a new trauma, most probably due to confusion, which is a well-known complication related to this drug [24]. Finally, patients treated, on discharge, with morphine only demonstrated the highest mortality, whereas those taking paracetamol demonstrated lower mortality. This observation is logical as it reflects the overall status of patient. In general, chronic and severe pain, which requires permanent opioid treatment, indicates advanced disease with poor prognosis, whereas the use of weak analgesics indicates a more benign disease course.

The reason that patients had in general more medications upon discharge than admission despite the medication optimization is related to the need for post-operative pain reduction (analgesics) as well as thrombosis prophylaxis (LMWH), both of which are routinely started at hospital and continued at discharge.

### Study strengths and limitations

Our catchment area is served by one university hospital, and all hip fractures were treated at the same study site. The hospital medical records were accessible and scrutinized by the researchers. Moreover, the Swedish record system is particularly appropriate for a study of this nature as the coverage of events of interest has high efficacy and validity. However, medical records from primary care were not available; thus, primary care physicians may have introduced some post-discharge changes in medications that were unnoticed by the authors.

## Conclusions

The total number of medications and use of specific drug classes are associated with increase readmission risk after acute hip fracture surgery in older patients. Medication reviews and withdrawal or modification of drugs may be justified in post-operative period as several drug-related problems were identified. Further studies, including randomized controlled trials on medication optimization, are needed.

## Abbreviations

ASA: American Society of Anesthesiologists
ATC: Anatomical Therapeutic Chemical
LMWH: Low-molecular weight heparin
SSRI: Selective serotonin-uptake inhibitor
VKA: Vitamin K antagonist
